# No evidence of subclinical infection in sheep surviving oral challenge with prions

**DOI:** 10.1099/jgv.0.002087

**Published:** 2025-03-21

**Authors:** M. Khalid F. Salamat, Nora Hunter, E. Fiona Houston

**Affiliations:** 1 Royal (Dick) School of Veterinary Studies, The Roslin Institute, The University of Edinburgh, Edinburgh, Midlothian, UK

**Keywords:** oral, prion, protein misfolding cyclic amplification (PMCA), sheep, subclinical

## Abstract

Variant Creutzfeldt–Jakob disease (vCJD) is a fatal zoonotic disease caused by the ingestion of bovine spongiform encephalopathy (BSE)-infected meat products. Although the number of vCJD cases due to dietary exposure has significantly declined, the true burden of subclinical infections remains uncertain. Several large-scale surveys using appendix tissue samples have indicated the presence of abnormal prion protein (PrP^Sc^; Sc for scrapie) in lymphoid tissue of a small proportion of the UK population. These may represent silent carriers of infection, with the potential to contribute to transmission, persistence and re-emergence of vCJD. Previously, we showed that subclinical infection is a frequent outcome of low-dose prion exposure by blood transfusion in sheep. To determine whether subclinical infection was also found following low-dose exposure by another clinically relevant route for humans, we screened archived tissues from sheep orally challenged with a range of doses of BSE, which did not show clinical or pathological signs of disease after several years of follow-up post-infection. Using a highly sensitive protein misfolding cyclic amplification assay, we were unable to detect PrP^Sc^ in the lymph node/tonsil of 15 sheep, or in a wider range of lymphoid tissues and brain (medulla oblongata) from a subset of 5 sheep. Our findings suggest that the route of infection/exposure may significantly influence the probability of establishing subclinical infection, with the oral route apparently much less efficient than intravenous infection by blood transfusion in sheep.

## Introduction

Transmissible spongiform encephalopathies (TSEs) or prion diseases are progressive fatal neurological disorders that affect both humans and animals [1]. They include bovine spongiform encephalopathy (BSE) in cattle, scrapie in sheep and goats, chronic wasting disease (CWD) in cervids and Creutzfeldt–Jakob disease (CJD) in humans with acquired, sporadic and hereditary forms. The hallmark of prion diseases is the misfolding and accumulation of a normal host-encoded prion protein, PrP^C^ (C for cellular form), into an aggregated form, PrP^Sc^ (Sc for scrapie), which represents the only current disease-specific marker [2]. Distribution of PrP^Sc^ aggregates in tissues of affected individuals depends on the host species, route of exposure and prion strain. In sporadic CJD and BSE, PrP^Sc^ mainly accumulates within central and peripheral nervous systems, whereas it is also found in secondary lymphoid tissues [lymph nodes, tonsils, spleen and gut-associated lymphoid tissue (GALT)] in scrapie, CWD and variant CJD (vCJD) [3,4]. Following peripheral exposure, a key step in the pathogenesis of most naturally occurring infectious prion diseases is prion replication in secondary lymphoid tissues before its spread to the central nervous system, termed ‘neuroinvasion’ [5].

Epidemiological evidence, strain typing and experimental transmission of BSE to non-human primates and transgenic mice expressing human PrP support the hypothesis that consumption of BSE-contaminated meat products led to the emergence of variant CJD in the UK population [6,9]. Measures to control the spread of classical BSE and limit human exposure have been very effective, and since 2012, only one case of variant CJD has been reported in the UK. To ascertain the true burden of BSE exposure in the UK population, several large-scale anonymized surveys using human appendix and tonsil samples have been conducted [10,11]. One such survey involving birth cohorts likely to have had dietary exposure to BSE estimated a prevalence of abnormal PrP in the appendix (lymphoid tissues) of up to 1 in 2,000 individuals [11]. There is a marked discrepancy between this projected figure and the number of confirmed vCJD cases (178) reported so far in the UK. Since millions of people potentially had dietary exposure to BSE during the epidemic, the appendix survey data raised concerns that a significant number of the healthy UK population might be ‘subclinical’ carriers of infection without developing overt clinical signs during their natural life span. Interestingly, transgenic mice expressing human PrP experimentally inoculated with BSE showed preferential prion replication in lymphoid tissues with limited neuroinvasion [12], suggesting that cross-species transmission barriers may not be equal for all tissues and providing a possible explanation for the observations of appendix surveys.

The role of subclinical infection in the persistence and natural transmission of prion diseases is not well understood, in part due to the lack of diagnostic tests that can reliably detect infected live animals. Studies using rodent models have shown that scrapie can persist and replicate throughout the host lifespan in the absence of clinical signs following cross-species transmission [13,14]. Other factors that contribute to the development of subclinical prion infection in laboratory models include dose and route of infection, host PrP^C^ expression levels, genotype and age, as well as specific immune deficiencies [15,18]. Several studies in which entire sheep flocks affected by natural scrapie were culled and examined have identified asymptomatic animals with PrP^Sc^ deposits in lymphoid tissues and/or brain, in some cases in animals up to 7 years old, which may be associated with variation in *PRNP* genotype and/or age at exposure [19,21]. However, in such studies, it is difficult to determine whether this represents preclinical or true subclinical infection, due to the lack of lifetime follow-up.

In a previous study of sheep exposed to low doses of BSE by intravenous blood transfusion, we demonstrated that a high proportion had low levels of PrP^Sc^ in lymphoid tissues, despite surviving for many years (in some cases close to their natural lifespan) without developing clinical signs [22]. We hypothesized that this apparent subclinical infection was due to a low infectious dose, and in this study, we aimed to determine whether subclinical infection also occurs following low-dose oral infection of sheep with BSE. To do so, we took advantage of archived tissues from clinically and pathologically negative sheep in a previous study that examined the age-related susceptibility of sheep to a range of oral doses of BSE [23].

## Methods

### Experimental infection of sheep

Tissue samples used in this study were from sheep used in a previously published study to determine the effect of age on susceptibility to oral infection with BSE [23]. Cheviot sheep were sourced from the Defra scrapie-free flock of New Zealand origin [24]. All sheep had the *PRNP* genotype ARQ/ARQ (denoting the amino acids encoded at codons 136, 154 and 171 of the *PRNP* gene). Further sequencing of a limited number of sheep was later carried out to determine the frequency of the P141L polymorphism, which is associated with variation in the incubation period in BSE-infected sheep [25].

Sheep inoculations were previously described in detail [23]. Briefly, groups of lambs or sheep of ages ∼24–48 h, 2–3 weeks, 3 months, 6 months and 15–27 months (adult) were orally infected with either 0.05, 0.5 or 1 g of BSE-infected cattle brain homogenate (BH) (containing 10^3.2^ ID_50_ per g after titration in RIII mice [26]), provided by TSE Archive, Animal Plant and Health Agency, Weybridge. All animals were monitored for the development of clinical signs of TSEs and were euthanized when they reached defined humane endpoints. Sheep that did not develop clinical signs were culled at the end of the study (>5.5 years post-challenge). A range of tissues (nervous and lymphoid) were collected from each sheep at necropsy and fixed in neutral buffered formalin or frozen immediately on dry ice and then stored at −70 °C. Brain and lymphoid tissue samples from each animal were tested for the presence of PrP^Sc^ by immunohistochemistry (IHC) and/or Western blot to confirm whether they were infected with BSE.

For this study, we selected five sheep from each of the groups challenged with 1 g BSE-infected BH at 3, 6 and 15–27 months of age. These sheep did not develop clinical signs of BSE and had negative post-mortem test results on tissues by IHC and/or Western blot and are referred to herein as ‘clinically negative, pathology negative’ animals.

### Protein misfolding cyclic amplification to detect PrP^Sc^

We used miniaturized bead serial protein misfolding cyclic amplification (PMCA) [27,28], a highly sensitive *in vitro* amplification assay (see [29] for detailed protocol) to detect PrP^Sc^ in tissues of the selected clinically negative, pathology-negative sheep. Briefly, 10% w/v BH from TgshpXI transgenic mice over-expressing sheep PrP^C^ from the ARQ *PRNP* allele was prepared in PMCA buffer (PBS pH 7.2, 0.25% v/v Triton X-100, 150 mM NaCl, Roche cOmplete Protease Inhibitor) at 4 °C for use as ‘substrate’. Tissues from BSE-challenged clinically negative, pathology-negative sheep, negative and positive controls (mock-infected and BSE-infected clinically positive, respectively) were homogenized in PBS, as previously described [22], to yield 10% w/v homogenates (termed ‘seed’). PMCA reactions were performed in Axygen 96 well PCR plates with 45 µl substrate and 5 µl seed per well, in the presence of one Teflon bead (2.381 mm, Marteau and Lemarie, France). Dextran sulphate (Sigma-Aldrich) was added to each reaction to give a final concentration of 0.5%. PMCA reactions seeded with tissue homogenates from clinically negative, pathology-negative sheep were run in triplicate. Positive controls run in each experiment included tenfold serial dilutions of prescapular lymph node and tonsil from an age-matched clinically positive BSE-infected sheep and pooled BH (BSB/7/10) from five BSE-infected pathology positive sheep. Negative control reactions were seeded with BH from mock-infected sheep to check for non-specific amplification.

Microplate-based PMCA reactions were performed in a microplate horn attached to a programmable Misonix Q700 sonicator (QSonica), using a water recirculation system to maintain temperature at 37 °C. Each PMCA round (24 h) consisted of 96 cycles of 10 s sonication (at an amplitude of 50–70%, with minimum output energy 4,000 W) and 14 min 50 s incubation. After one round, one-tenth of the reaction mixture was added to the fresh substrate to seed the next round. Following two rounds of amplification, part of each reaction (18 µl) was digested with proteinase K and immunoblotted for detection of PrP^Sc^, using ROS-BC6 as the primary antibody (0.25 µg ml^−1^), as previously described [29].

## Results

In the previous study [23], groups of sheep ranging in age from newborn lambs to adults were infected with 0.05, 0.5 or 1 g BSE-infected cattle BH by oral (intrabuccal) dosing. In the youngest age groups (<2 days old, 2–3 weeks old), the majority of animals developed clinical BSE regardless of the dose received, whereas in older age groups (3 months, 6 months, ≥15 months), a high proportion of exposed individuals survived for >2,000 days post-infection without developing clinical disease or showing pathological evidence of infection by IHC or Western blotting (Tables 1 and S1, available in the online Supplementary Material). The latter group, termed ‘clinically negative, pathology negative’, was, thus, comparable to the long-term survivors of BSE-infected blood transfusions that previously showed evidence of subclinical infection [22].

**Table 1.** PMCA testing of tissues from sheep orally exposed to 1 g BSE-infected cattle BH

Age at challenge	Disease status	No. of sheep	Survival period(days post-infection)	PMCA testing results (no. of positive results/no. of tissues tested)					
				PSLN (LoD*)	Tonsil (LoD)	IPP	MLN	Spleen	Brain
3 months	BSE negative	10†	217–2,143	0/5	0/5	nt	nt	nt	nt
6 months	BSE positive	4	652–847	1/1 (10^−6^–10^−9^)‡	1/1 (10^−6^–10^−7^)‡	nt	nt	nt	nt
	BSE negative	6	682–2,062	0/5	0/5	0/5	0/5	0/5	0/5
≥15 months (adult)	BSE positive	2	930–1,010	1/1 (10^−6^)§	1/1 (10^−7^–10^−8^)§	nt	nt	nt	nt
	BSE negative	8	1,359–2,297	0/5	0/5	nt	nt	nt	nt

* LoD=limit of detection, i.e. last tenfold dilution positive for PrPSc amplification after two rounds of PMCA.

† None of ten challenged animals tested positive by immunohistochemistry or Western blot.

‡ Range shows variation in LoD in five different PMCA experiments.

§ Range shows variation in LoD in two different PMCA experiments.

IPPileal Peyer’s patchMLNmesenteric lymph nodeNTnot tested

**Fig. 1.**
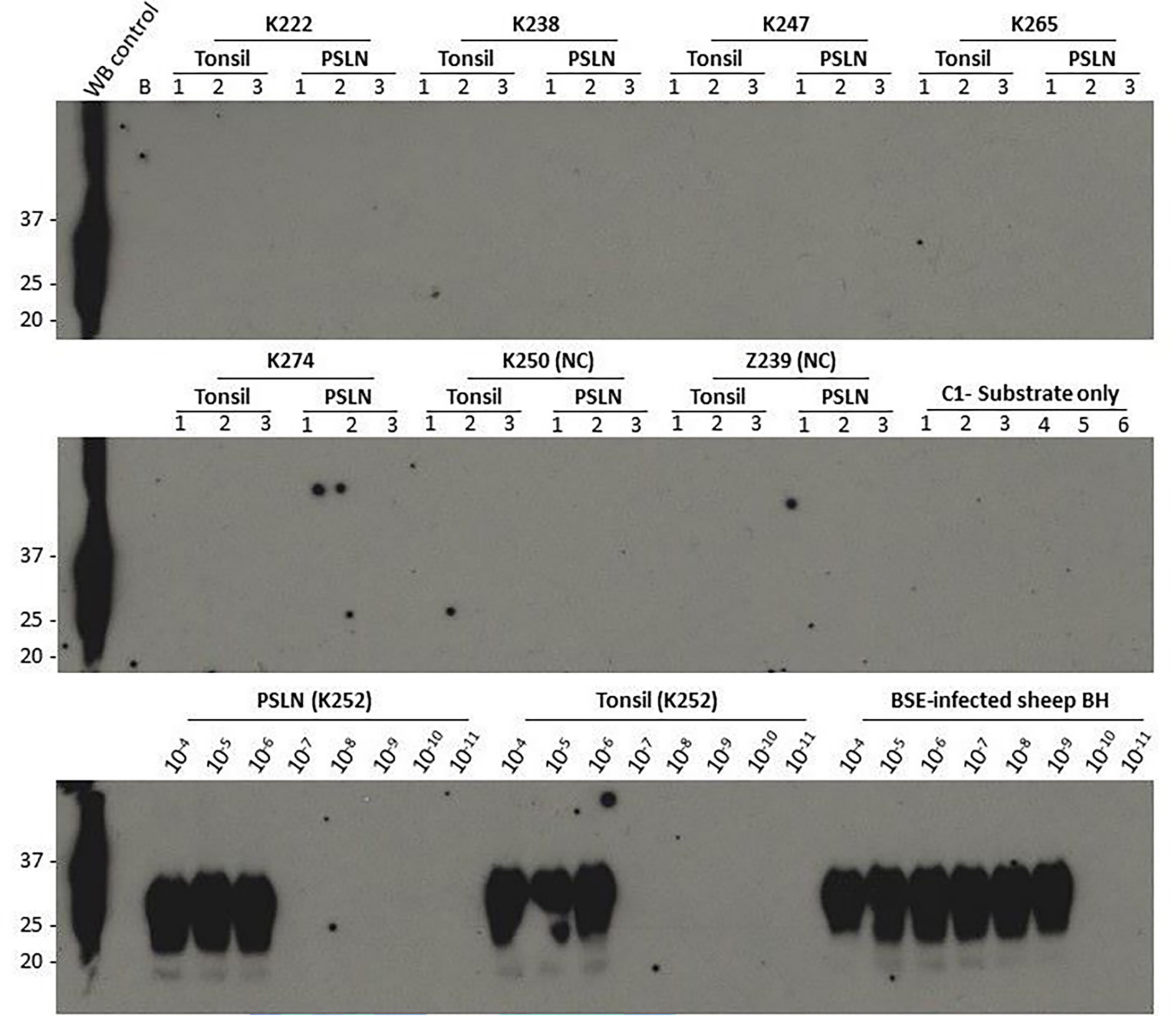
PMCA results for tonsils and prescapular lymph node (PSLN) of clinically negative, pathology-negative sheep challenged with BSE at 6 months of age. Western blot of PK-digested PMCA reaction products for tissue samples (run in triplicate) from individual sheep (indicated by their ID numbers). WB control; un-infected ARQ sheep BH (not PK digested; 1.7 mg brain equivalent). B – blank lane. Negative controls were run on the same plate. *Negative controls*: C1 – substrate only (run in six replicates), NC=negative controls: K250 – mock-infected sheep at 6 months of age, Z239 – mock-infected sheep at 28 months of age. *Positive controls*: Tenfold dilutions from PSLN and tonsil from a clinically positive sheep (K252) from the same age group at exposure (6 months) and brain homogenate pool (BSB/7/10) from clinically positive BSE-infected sheep.

Tissues from five ‘clinically negative, pathology negative’ sheep in each age group (3 months, 6 months, ≥15 months), dosed with 1 g BSE-infected brain, were selected for analysis using a sensitive method for PrP^Sc^ detection, PMCA. Our previous experiments in sheep exposed to BSE by blood transfusion showed that subclinical infection was most consistently detected in pre-scapular (superficial cervical) lymph nodes (PSLN). In orally infected sheep, we expected that the tonsil would be one of the first sites of BSE replication and accumulation. Therefore, tonsil and PSLN samples from the selected animals were initially screened by PMCA; however, all samples tested negative (Tables 1 and S1). By contrast, with serial tenfold dilutions of equivalent tissues from clinically positive sheep dosed with 1 g BSE-infected brain, positive PMCA reactions were obtained to dilutions of 10^−6^–10^−9^ for PSLN and 10^−6^–10^−7^ for tonsil (Fig. 1 and Table 1).

PMCA analysis was then extended to a greater range of tissues (ileal Peyer’s patch, mesenteric lymph node, spleen, brain – medulla oblongata) in the group of sheep orally infected with BSE at 6 months old. We reasoned that since this group had the highest number of clinical BSE cases (4/10), there was a greater probability of detecting subclinical infection in the long-term survivors. However, there was no evidence of positive amplification of PrP^Sc^ in any of the tested tissues following two rounds of PMCA (Table 1).

## Discussion

One of the major challenges in the control of infectious prion diseases in animals and humans is the long asymptomatic incubation period, during which disease can be spread through various transmission routes and by movements of infected individuals. This preclinical phase of the disease can last for years or even decades, making it difficult to distinguish from subclinical infection (defined as infections that will not result in disease within the natural lifespan of the species). Although subclinical prion infection has been documented in laboratory animals, its prevalence and potential contribution to transmission, persistence and re-emergence of prion diseases in natural hosts are poorly understood. From rodent experiments, factors that appear to contribute to the establishment of subclinical infection include cross-species transmission, low infectious dose, route of infection, age and immune status [13,18]. In a previous study, we showed that a high proportion of sheep exposed to low doses of BSE by intravenous transfusion of blood components had very low levels of PrP^Sc^ in their tissues (detectable only by ultrasensitive PMCA) up to 10 years post-infection [22]. As these individuals were apparently healthy and close to the average natural lifespan for sheep (12–14 years), this most likely represented true subclinical infection. By contrast, in the current study, we were unable to detect PrP^Sc^ by PMCA in lymphoid tissues and brain (medulla oblongata) of sheep orally exposed to low doses of BSE, which survived up to 6 years post-infection without clinical or pathological evidence of disease. Although the number of sheep tested was relatively small (15 for PSLN/tonsil; 5 for additional lymphoid tissues and medulla oblongata), they should be sufficient to detect subclinical infection at the levels seen in the transfusion study [22].

Our results demonstrate that the route and method of infection have a major effect on the probability of establishing subclinical BSE infection in sheep. The infectious doses received by each route cannot be directly compared, but based on previous titration of BSE-infected sheep brain and blood in transgenic mice expressing bovine PrP [30], the dose given to orally infected sheep would have been ~3–4 log greater than the dose from transfusion of 450 ml of infected whole blood. However, the source of inoculum (BSE-infected sheep blood vs. BH) may also have an influence on the likelihood of establishing subclinical infection. Indeed, a previous study demonstrated that transfusion of live white blood cells from scrapie-infected sheep resulted in much more efficient transmission of disease than scrapie-infected BH administered intravenously [31].

One of the major factors determining the probability of infection (clinical or subclinical) following oral exposure to prions is likely to be the efficiency of uptake in the gastrointestinal tract. This will be influenced by the extent to which normal digestive processes and gastrointestinal microflora contribute to the inactivation of prion infectivity before it reaches potential sites of uptake (Peyer’s patches and other GALT). The age at infection has a marked effect on the susceptibility of sheep to oral infection with BSE, potentially related to postnatal changes in gut permeability, microbiome (during transition to rumination) and maturation/involution of GALT, as discussed in detail previously [23]. A number of studies have demonstrated that exposure of BSE or scrapie isolates to conditions simulating rumen fermentation does not result in significant degradation of PrP^Sc^ [32,33], while incubation with sheep small intestinal contents produced very variable degradation [34]. These studies did not determine whether the persistence of PrP^Sc^ was accompanied by retention of infectivity. In addition, dilution of orally administered inocula in ruminal contents, and the effect of rumination processes on transit times of ingesta, may influence the rate of delivery of infectivity to the small intestine and, thus, the likelihood of establishing infection in Peyer’s patches. Therefore, it seems probable that conditions for establishing subclinical infection following oral ingestion of prions are very different in ruminants and monogastric animals such as laboratory rodents and humans.

Experiments in wild-type mice have shown that subclinical infection can follow oral administration of a dose of mouse-adapted scrapie that induced clinical disease when administered by intracerebral or intraperitoneal routes. Interestingly, levels of PrP^Sc^ and infectivity in the brain were similar in subclinical and clinical mice challenged by different routes, but infectivity levels were comparatively low in the spleens of subclinically infected animals [15].

Two studies have demonstrated subclinical infections of ruminants following an experimental challenge by the oral route. In four out of five white-tailed deer orally inoculated with urine and faeces from CWD-infected deer, which remained healthy 19 months post-infection, PrP^Sc^ was detected by PMCA in the obex (brainstem) only but not in lymphoid or other neural tissues [35]. Five goats challenged orally with 5 g BH from an atypical l-BSE case survived up to 6.5 years post-infection without clinical signs, and PrP^Sc^ could be detected in the brain, lymphoid tissues and spinal cord using real-time quaking-induced conversion (a PrP^Sc^ amplification technique similar to PMCA) [36]. In both studies, post-mortem tests on tissues for PrP^Sc^ using conventional assays such as IHC, Western blot and ELISA gave negative results, showing that the levels of PrP^Sc^ present were very low. It is difficult to determine precisely the reasons for differences between these studies and our current results in sheep orally challenged with classical BSE, given differences in species, age at challenge, prion agents, types of inocula and doses used.

Polymorphisms of the *PRNP* gene encoding PrP^C^ are a major determinant of host susceptibility to prion diseases and may also be a factor influencing the establishment of subclinical infections [37,38]. The sheep used in this study had the *PRNP* genotype ARQ/ARQ (where each letter indicates the amino acid encoded at codons 136, 154 and 171), selected because it was associated with the greatest susceptibility to experimental BSE challenge [39]. Subsequent sequencing of a subset of the animals revealed the presence of a polymorphism at codon 141 (P141L), which was associated with variation in incubation periods but not susceptibility to BSE [23,25]. Although codon 141 *PRNP* genotypes were not determined for all of the clinically negative, pathology-negative sheep screened by PMCA in this study (see Table S1), the lack of association between codon 141 genotype and BSE susceptibility suggests that it is unlikely to explain the failure to detect PrP^Sc^ in these animals.

In summary, in contrast to our findings in sheep exposed to low doses of BSE by blood transfusion, we did not find any evidence of subclinical infection in long-term survivors of oral challenge with BSE. This most likely reflects the respective efficiencies of the different routes of infection and differences in the source of infectivity (blood versus brain) and highlights the potential importance of host physiology in determining outcomes of infection.

## supplementary material

10.1099/jgv.0.002087 Uncited Table S1.

## Abbreviations

BH: brain homogenate
BSE: bovine spongiform encephalopathy
CJD: Creutzfeldt–Jakob disease
CWD: chronic wasting disease
GALT: gut-associated lymphoid tissues
PrPC: prion protein cellular form
PrPSc: prion protein scrapie
TSEs: transmissible spongiform encephalopathies
vCJD: variant Creutzfeldt–Jakob disease
